# Gender and Sexual Health-Related Knowledge Gaps and Educational Needs of Parents of Transgender and Non-Binary Youth

**DOI:** 10.1007/s10508-023-02611-9

**Published:** 2023-05-26

**Authors:** Lane Z. Kantor, Diana M. Tordoff, Samantha G. Haley, Julia M. Crouch, Kym R. Ahrens

**Affiliations:** 1 David Geffen School of Medicine at the University of California Los Angeles, 10833 Le Conte Ave., Los Angeles, CA 90095 USA; 2 Department of Epidemiology, Stanford University School of Medicine Palo Alto, Stanford, CA USA; 3 Woodinville Pediatrics, Woodinville, WA USA; 4 Center for Child Health, Behavior and Development, Seattle Children’s Research Institute, Seattle, WA USA; 5 Department of Pediatrics, School of Medicine, University of Washington, Seattle, WA USA

**Keywords:** Transgender, Non-binary, Parents, Sex education, Sexual health, Gender dysphoria

## Abstract

Parents can have a substantial impact on adolescent sexual decision-making, but few studies have explored the role of parents in providing sexual health information to transgender and non-binary (TNB) youth, a population that experiences significant sexual and mental health disparities and lower levels of perceived family support compared with other youth. This study aimed to elucidate existing knowledge gaps and content deemed important for a sexual health curriculum and educational materials for parents of TNB youth. We conducted 21 qualitative interviews with five parents of TNB youth, 11 TNB youth aged 18+, and five healthcare affiliates to identify parents’ educational needs. We analyzed the data using theoretical thematic analysis and consensus coding. Parents self-reported multiple knowledge deficits regarding gender/sexual health for TNB individuals and were primarily concerned with long-term impacts of medical interventions. Youth goals for parents included greater understanding of gender/sexuality and sufficient knowledge to support youth through social transition to their affirmed gender identity. Content areas suggested for a future curriculum for parents of TNB youth included: basics of gender/sexuality, diverse narratives of TNB experiences/identities, gender dysphoria, non-medical gender-affirming interventions, medical gender-affirming interventions, and resources for peer support. Parents desired access to accurate information and wanted to feel equipped to facilitate affirming conversations with their child, something that could combat health disparities among TNB youth. An educational curriculum for parents has the potential to provide a trusted information source, expose parents to positive representations of TNB individuals, and help parents support their TNB child through decisions about potential gender-affirming interventions.

**Supplementary Information:**

The online version contains supplementary material available at 10.1007/s10508-023-02611-9.

## Introduction

Transgender and non-binary (TNB) youth experience barriers to accessing comprehensive and inclusive sex education (Bradford et al., 2019; Haley et al., 2019; Tordoff et al., 2021). In a representative study of 3038 youth aged 12–24, adolescents identified their parents as being the most influential people in shaping their sexual decision-making—including age of sexual debut and use of contraception, condoms, and sexually transmitted infection (STI) testing—with 52% of youth aged 12–15 and 32% of youth aged 16–19 reporting parents as the most influential factor (Guilamo-Ramos et al., 2020; Power to Decide, 2016). Only 17% of youth aged 12–15 and 28% of youth aged 16–19 reported that their friends were the most influential people in shaping their sexual decision-making (Power to Decide, 2016). However, few studies have explored the role of parents in providing sexual health information to TNB youth specifically. This area of research is particularly important given the evidence that transgender adolescents are at higher risk of acquiring HIV and other STIs and are more likely to report the use of drugs or alcohol before last sexual intercourse (Garofalo et al., 2006; Herbst et al., 2008; Johns et al., 2019; Reisner et al., 2019). This research is also particularly essential and salient at this moment, given escalating legislative attacks on LGBTQ-affirming content in schools and access to gender-affirming care for TNB youth (Barbee et al., 2022; Kremen et al., 2021).

Studies have shown that parental responses to their child’s lesbian, gay, or bisexual identity correlate with mental and physical health outcomes for the child (Huebner et al., 2013). It has been demonstrated that some parents experience and display strong negative emotional reactions when their child first comes out as LGBTQ, which can have a profoundly negative impact on their child’s well-being. Negative parental reactions are correlated with increased risk of suicide attempts and substance misuse among sexual minority youth (Beeler & Diprova, 1999; Ryan et al., 2009; Saltzburg, 2004) and LGBTQ youth more broadly (Newcomb et al., 2019, 2020).

Conversely, there is also substantial research to suggest that family support is a protective mechanism for TNB youth with respect to a wide range of health disparities (Bockting et al, 2013; Grossman et al., 2006, 2021; Olson et al., 2016; Ryan et al., 2010, Valente et al., 2020). The literature on TNB youth has documented elevated rates of poly-victimization (such as bullying, violence, and family rejection), suicidality, anxiety, and depression among TNB youth compared to their cisgender peers (James et al., 2016; Newcomb et al., 2020; Olson et al., 2015; Reisner et al., 2015; Wilson et al., 2009). However, family connectedness and parental support has consistently been associated with improved outcomes across many of these domains for TNB youth, including lower rates of depression, suicidal ideation, disordered eating, PTSD, and perceived burden of being transgender, as well as improved self-esteem, quality of life, and reports of increased condom use during sex (Andrzejewski et al., 2021; Bauer et al., 2015; Johns et al., 2018; Olson et al., 2015; Simons et al., 2013; Veale et al., 2017; Watson et al., 2016; Wilson et al., 2012, 2016). Further, the National Transgender Discrimination Survey found that compared to those with unsupportive families, transgender respondents with family support were less likely to have ever engaged in sex work, experienced homelessness, or attempted suicide (James et al., 2016). A number of these studies have called for more research into specific interventions that will promote parental acceptance and support.

Many parents of LGBTQ youth desire access to information that will help them support their child’s health and well-being (Mustanski et al., 2017; Newcomb et al., 2019, 2020). Even among this group of parents who intend to be supportive, many lack the knowledge and skills necessary to help their LGBTQ child navigate future dating and sexual relationships (e.g., due to limited knowledge of STIs and safer sex practices specific to LGBTQ individuals) (Mustanski et al., 2017; Newcomb et al., 2019, 2020).

Finding useful and accurate sexual health information as a parent may be particularly difficult for parents of TNB youth, and a lack of resources may exacerbate parental feelings of helplessness and confusion. An international literature review by Sharek et al. (2018) examined the experiences of transgender young people and their families and found that their families experienced extensive difficulty accessing information about transgender identities. Family members’ ability to make sense of their loved one’s transgender identity was correlated with their ability to access appropriate, useful, and relevant information on this topic. Even among families who described finding information that improved their ability to support their child, most families still felt there were insufficient educational resources available to them. This lack of information often exacerbated parental feelings of powerlessness, which sometimes led to parents envisioning worst case scenarios for their child.

When resources were available to family members, self-education was seen as an active and positive coping strategy for confusion, anger, and other initial negative emotions that arose after their loved one came out to them (Sharek et al., 2018). Most family members accessed information through the internet, although some learned about gender identity directly from their transgender family member or from healthcare professionals. Healthcare professionals were described as helpful in facilitating conversations, developing parents’ understanding of their child’s gender identity, and brainstorming how they could best support their child. Education and accurate information helped parents feel more secure in their ability to parent a transgender child, less guilty or confused about supporting their child’s choices, and more confident in their ability to make the best decisions for their child moving forward. Gaining knowledge was also associated with helping parents feel less isolated and alone. Another notable finding from this review was that access to information and education was instrumental in allowing family members to move beyond their individual lens (e.g., simply accepting and supporting their personal family member) toward broader empowerment to advocate for their family member and other TNB people.

Additionally, most sources of puberty and sexual health information utilize binary and cisnormative language about bodies and often exclude information on non-normative genders and sexualities (Riggs & Bartholomaeus, 2018). Thus, TNB youth and their parents face challenges in accessing information on these topics that is both medically accurate and inclusive of TNB people. One study of online resource use by transgender youth and their parents found that while looking for information, youth and parents often encountered blatant misinformation and hate speech against transgender people (Evans et al., 2017). Many parents in the study discussed internet research as being risky and that some online articles were laced with malicious messaging against TNB persons. Because of these challenges, it is important that parents of TNB youth are able to access accurate sexual health information relevant to the experiences of TNB youth and that they know how to discuss it with their children in an affirming manner.

Though it is clear that familial knowledge, education, and support is important for TNB youth, few studies have specifically examined the needs of parents raising TNB children. One study examined the experiences of parents raising TNB children and found that the most common parental needs were for information (research and guidelines), social support, and educational resources to provide to schools, peers, professionals, and their local community (Riley et al., 2013a). Parents in this study believed that access to information and resources would provide them with skills to counteract bullying and negativity toward themselves and their children, as well as help them tolerate the uncertainty of not definitively knowing future outcomes for their child (Riley et al., 2013a).

Another study assessed the retrospective childhood experiences of transgender adults and found that these adults wished their parents had increased access to information about transgender identities and experiences in order to assist in conversations with other family members, teachers, and parents. The study also found that these transgender adults believed their parents would have benefitted from access to peer support and educated health professionals (Riley et al., 2013b). A study examining the success of an educational and therapeutic support group for parents of transgender youth also found that active parent participants were more likely to report improved parental functioning, harmonization of the parent–child relationship, peaceful family dynamics, and improved adolescent behavior (Boivin et al., 2020). However, to our knowledge, no study has assessed explicit content areas that TNB youth and parents deem important for parents.

To fill this literature gap, we aimed to identify needed content areas with respect to gender, sexuality, puberty, and sexual health for parents raising TNB youth. This is a secondary analysis of a qualitative study that aimed to understand deficits in sex education content experienced by TNB youth and to identify content and best practices for a comprehensive and trans-inclusive curriculum for TNB youth and their families. Two prior sub-analyses explored the sexual health education needs of TNB youth. The first assessed prior sex education experiences of TNB youth and recommended curricular content for a gender and sexual education program tailored to TNB youth (Haley et al., 2019). The second explored the importance of employing transgender-inclusive language during the delivery of sexual health content for TNB youth (Tordoff et al., 2021). The analysis presented here shifts the focus to parental perspectives and aims to identify gaps in sources of sexual health information available to parents of TNB youth and explore content deemed necessary or desirable to include in a curriculum for parents of TNB youth.

## Method

### Participants

A total of 21 interviews were conducted among five parents of TNB youth, five healthcare affiliates associated with the Seattle Children’s Gender Clinic (SCGC), and 11 youth aged 18 or older, including six transmasculine, three transfeminine, and two non-binary individuals. For the purposes of this study and recruitment materials, we used the term transgender as an umbrella term to describe a diverse group of people who have a gender identity that differs from the sex they were assigned at birth. The term non-binary refers to individuals with gender identities that exist outside binary notions of gender (male/female, man/woman, and boy/girl) and may identify as neither male nor female, both male and female, no gender at all, or as different genders at different times. Youth and parent participants were recruited in-person at Seattle Children’s Gender Clinic (SCGC) by posting fliers and study announcements throughout the clinic as well as through regional listservs of transgender community organizations.

Youth participants ranged from 18 to 26 years old. We intentionally recruited TNB youth spanning late adolescence into young adulthood (ages 18–26) to capture a range of perspectives, from impressions after recently experiencing sex education to reflections after a few more years of lived experience. Existing literature on parenting TNB youth focuses primarily on the perspectives and experiences of parents and caregivers; therefore, we intentionally recruited more youth than parent participants. As the recipients of the information and the individuals with the lived experience of being TNB, we consider the youth perspectives on what they believe their parents should know to be just as valuable as the perspectives of the parent participants.

All parents interviewed were cisgender women, including four parents of transmasculine children and one parent of a non-binary child, whose children’s ages ranged from 14 to 24 years old. Although efforts were made to include male parents/caregivers, all who were approached were not interested or unavailable to participate in the study. The parents of TNB youth interviewed in this study were not parents of the interviewed TNB youth. This methodological decision was made to maximize the likelihood that youth and parents could speak honestly about their experiences without being concerned about what their child/parent may separately say to the same interviewer.

Lastly, as practitioners working with both TNB youth and their families, the healthcare affiliates provided a unique and important perspective that was not provided by youth and parents alone. Healthcare affiliates were identified by the authors, contacted directly, and sampled to be representative of the major care roles at SCGC. Healthcare affiliates included three physicians (an adolescent medicine specialist, a pediatric endocrinologist, and a pediatric primary care physician), a licensed clinical social worker, and a patient advocate who serves on the community advisory board for SCGC. All interviewed youth and parent participants were white (Table 1).

**Table 1:** Demographic characteristics of youth and parent participants

ID	Participant type	Gender of participant	Age of participant	Gender of child	Age of child	Recruitment method
1	Youth	Non-binary	26	–	–	Community listserv/flier
2	Youth	Transgender man	20	–	–	Community listserv/flier
3	Youth	Non-binary	19	–	–	Community listserv/flier
4	Youth	Transgender woman	20	–	–	Community listserv/flier
5	Youth	Transgender man	18	–	–	SCGC
6	Youth	Transgender woman	18	–	–	SCGC
7	Youth	Transgender man	18	–	–	SCGC
8	Youth	Transgender man	18	–	–	SCGC
9	Youth	Transgender man	18	–	–	SCGC
10	Youth	Transgender man	18	–	–	SCGC
11	Youth	Transgender woman	19	–	–	SCGC
12	Parent	Cisgender woman	50	Non-binary	24	Community listserv/flier
13	Parent	Cisgender woman	47	Transgender man	18	Community listserv/flier
14	Parent	Cisgender woman	48	Transgender man	17	SCGC
15	Parent	Cisgender woman	58	Transgender man	14	SCGC
16	Parent	Cisgender woman	51	Transgender man	14	SCGC

ID	Participant type	Role at SCGC	Recruitment method
17	Healthcare affiliate	Adolescent medicine physician	Contacted by investigator via email
18	Healthcare affiliate	Pediatric endocrinologist	Contacted by investigator via email
19	Healthcare affiliate	Pediatric primary care physician	Contacted by investigator via email
20	Healthcare affiliate	Clinical social worker	Contacted by investigator via email
21	Healthcare affiliate	Patient advocate	Contacted by investigator via email

### Procedure

We conducted 30–60 min qualitative, semi-structured interviews in order to identify the sexual health education needs of TNB youth and their parents. Interviews with parents and youth were conducted either in-person or via phone based on participants’ preferences. In-person interviews were conducted at a preferred location for TNB youth and parents (i.e., a café, their home). Interviews with healthcare affiliates were conducted in-person in the healthcare affiliate’s office. All interviews were conducted by one researcher (DMT), a white cisgender woman and doctoral student with over five years of experience working with LGBTQ youth.

An interview guide was developed, and additional probes were added throughout the interview process based on what came up for participants. Interview questions varied based on participant type, and example prompts are available in Supplement 1. We asked questions about the following topics: sources of information about sexual health, parent–child conversations about sexual health, knowledge gaps and desired content areas for a future curriculum, the importance of having access to sexual health information, healthcare affiliate interactions with patients and their parents, and desired modes of delivery for a curriculum (Table 1). All parent and youth participants who completed the interview received $20; healthcare affiliates were not compensated for their time. All interviews were completed prior to beginning the coding and analysis phase. Interviews continued until the interviewer detected an acceptable degree of consistency/saturation on major topic areas using methods of theoretical saturation (Sandelowski, 1995; Saunders et al., 2018). We acknowledge that saturation end points in qualitative research are inherently subjective and influenced by both participant and interviewer identities; thus, a single study should never be assumed to provide the “whole picture” on a given qualitative topic (see additional discussion on this Limitations section). The methods for this study have been previously described in more detail (Haley et al., 2019; Tordoff et al., 2021).

### Coding and Analysis

Theoretical thematic analysis techniques were used to analyze the transcribed interviews and to identify patterns and themes within qualitative data (Braun & Clarke, 2006). A codebook of main subthemes was developed, and consensus coding was conducted by three independent researchers (LZK, DMT, and SGH) in Atlas.ti Version 8. We created the preliminary codebook based on prior research, clinical experience, and the interview guide. Next, members of the analysis team each independently reviewed and coded a sample of three (14%) exemplar interview transcripts, discussed and compared coding, and refined the preliminary codebook. After the development of the preliminary codebook, each coder independently read the remaining (*n* = 19, 86%) transcribed interviews and applied existing codes to the text. Additional codes were added to the codebook using an inductive approach to identify new themes based on the data. Coders also made comments in the master codebook to create, collapse, or refine theme definitions; modifications to the codebook were documented to ensure accuracy/consistency in the application of the codes. After transcripts were independently coded, annotated transcripts were combined, and each code application was jointly reviewed by all three coders to ensure consensus. Every difference in coding was discussed among all three coders until consensus was obtained. Participant categories (e.g., youth, parents, and healthcare affiliates) were initially analyzed separately, then subsequently compared and contrasted. All three coders have clinical experience working with TNB youth and include a white non-binary medical student, a white cisgender doctoral student, and a white cisgender pediatrician.

## Results

Three main themes emerged in discussion with participants: (1) the importance and perceived effectiveness of parent–child conversations about sexual health, puberty, and gender, (2) perceived barriers to parents accessing trusted sources of information, and (3) deficits in gender and sexual health knowledge relevant for TNB youth, with corresponding desired curricular content for parents. Table 2 summarizes themes and exemplar quotes.

**Table 2:** Themes, descriptions, and exemplar quotes

Theme	Description	Exemplar quote
Parent–child conversations	In contrast to parent-led discussions of cisgender puberty and anatomy, youth often felt frustrated by perpetually filling the role of teacher in conversations with their parents about gender	“It’s important for parents and other adults in children’s lives to understand [transgender health], and how [the parent] can be supportive, so that youth don’t have to bear the burden of doing all the emotional labor of educating everyone in their lives when they’re still figuring this out.” (ID3)
Perceived barriers to accessing information	Many parents struggled to find credible sources of information about gender identity and medical/surgical interventions for transgender people. This exacerbated knowledge imbalances between youth and parents	“When he started saying ‘I want to go on T [i.e., testosterone]; I want to have top surgery’ then we were like, "Where’s the research?" And it was incredibly hard to find… I was literally at the library, trying to look up medical articles, and I can hardly read them. But I just wanted to know, what are the side effects, just kind of the basic stuff before I’d even gotten to talk to a doctor.” (ID15)
Curricular content: Gender identity and trans-competence	All participants described the need for a curriculum to address basic concepts about gender identity and sexual orientation. Youth and providers emphasized dispelling myths about gender identity being a choice or a “phase”	“I think the biggest [myth] is that people think this is a choice that a kid makes, to be trans…They’re thinking that you’ve got a teenager [that] just decided one day that they wanted to be trans. I think a lot of parents who don’t accept that their kid is trans, it’s because they feel that their child has decided they want to be this, and it’s just a fad that’s going to pass. I mean, I wondered that too when we first faced this, but I’ve since learned that no, that’s not the way it goes.” (ID16)
Curricular content: Diverse narratives	Youth and parents recommended exposure to a diverse set of narratives and representations of TNB individuals and experiences as a way to lessen concerns about future obstacles and hardships, as well as normalize TNB people	“[When I hear] stories about people who don’t identify as one of the binary sexes, male or female, and these people sharing personal stories… [it] give[s] me insight as to how my child might be feeling. And also, it helps me understand better how to respond to people who don’t identify in the traditional way we've been taught to see gender. And I think that’s incredibly helpful too because… we’re not our sexuality. We’re people, and our sexuality is one part of our identity. And people sharing their stories about how it is to be seen as different and how they perceive barriers, the things that make them sad, the things that cause them pain, and then how they triumph through those challenges and find people who support them. I really enjoy those stories because it gives me hope that my own child's going to be okay.” (ID15)
Curricular content: Dysphoria	Participants wanted parents to understand gender dysphoria, the exacerbating effects of puberty, and the importance of gender-affirming techniques for mitigating the impact of dysphoria	For a lot of people, the case is that puberty worsens gender dysphoria, right? So helping parents understand that and how that might fit into their child’s story when [parents say], “Oh, I don’t know how suddenly, two years ago, their grades dropped, and they got really upset, and they lost all their friends, and also puberty.” And I’m like, “Okay, how do we connect those dots?” Because if you’re not looking for it, you don’t. (ID17)
Curricular content: Non-medical gender-affirming interventions	Participants recommended the inclusion basic information about non-medical gender-affirming techniques (such as binding and tucking), their importance to many TNB people, and their relevant safety concerns	“When I was binding unsafely, my mom didn’t really know about binding safety. So if I said, ‘No, it’s fine,’ she just had to take my word for it. Which was convenient for me, if I wanted to keep binding, but for safety reasons, I think it’s important that parents can access the information on their own, just because not everyone else is going to be upfront about it. And it’s easier for my mom to point out to me that I need to stop binding because it's not safe than for me to make that decision on my own.” (ID10)
Curricular content: Medical gender-affirming interventions	Participants recommended information about medical gender-affirming interventions, such as puberty blockers, HRT, and surgical options. Parents were most concerned about possible long-term complications, while youth were most concerned that their parents had enough information to support them as they navigated the medical system	“It would be really helpful just to have the basics of [HRT]… this is information the kid is likely to have well before the parent and the parent will be catching up on. So having some basic information in there about how [exogenous hormones] work, and what potential ups and downs are, pros and cons, and things that you need to think about – decisions you need to make together.” (ID15)
Peer support for parents	Opportunities for peer support were touted as an important way for parents to seek out advice, process complex emotions, build confidence in leading conversations about TNB-related topics, and feel less alone in the experience of parenting a TNB child	“…[T]here’s a place for [parents] to be validated that it’s a common experience to have grief. It’s a common experience to feel disoriented… and saying everyone’s experience is going to be different, but here are some things that other parents of trans kids have experienced that you might find reassuring to know that you’re not alone in this.” (ID12)

### Parent–Child Conversations

Every parent and youth participant described at least one parent–child conversation about sexual health and puberty, with significant variation in the content covered and perceived effectiveness of the conversation. Participants characterized these conversations relative to whether or not the youth had already disclosed their TNB identity to their parent.

#### Conversations Prior to Coming Out

Every parent described some conversation prior to their child coming out as TNB, often before their child began puberty: “It’s important to start talking about their sexual health when they’re young and start to be curious about their bodies and why they have the parts that they do” (ID14). During these “pre-coming out” conversations about puberty, parents assumed their child would experience a puberty that was aligned with their sex assigned a birth (sometimes described by youth participants as “cisgender puberty”). In addition to verbal conversations, some parents recalled providing books, pamphlets, and other written materials to their child. Common goals described by parents during these conversations were approachability, honesty, use of clear and anatomical language, and using the child’s curiosity as a cue to offer further information. Several parents recounted teaching their children to use anatomical terms for their body parts. One parent shared, “We call body parts what they are, we didn’t come up with little nicknames for them” (ID12). Most parents tried to create a level of comfort that encouraged their child to approach them with questions and concerns: “It was always our goal with our children to establish a relationship where they could come to us with questions” (ID14).

Prior to their child coming out, parents focused on providing information about general sexual health topics, particularly around the basics of puberty and anatomy, and occasionally on topics related to pregnancy and STI prevention. None of the parent participants reported discussing gender identity with their children prior to the youth coming out as transgender. There was a thematic tension across interviews between parent and youth participant themes on this topic. Parents described aiming to present themselves as approachable for their children’s questions and concerns. In contrast, youth participants expressed a wide range of comfort levels when discussing sexual health topics with their parents.

Youth participants recounted that parent–child conversations were often a source of basic puberty education, though these conversations were often felt to be insufficient for their needs. One youth shared that they had “some fairly limited conversations about sex and puberty” with their parents (ID1). Many youth participants felt that their parents did not do an adequate job providing them with information. Others experienced general discomfort or embarrassment talking to their parents about puberty and sexual health. These youth shared that discussing sex with parents simply felt “weird,” whereas for others, undercurrents of chronic parent–child tension made openness on an intimate topic like sex unthinkable. One participant summarized, “I didn’t have a very functional relationship with much of my family, so they probably weren’t the best people to ask” (ID2). Most youth described obtaining the majority of their sexual health and puberty knowledge from peers and online sources, with few youth participants believing they received useful information in school or through parent–child conversations.

#### Conversations After Coming Out

Among youth who were out to their parents as transgender, many described having to teach their parents about gender and sexuality-related topics. Parent participants in this situation usually described this as a positive, comfortable dynamic, and also believed that their child explicitly did not want to be taught by a parent:

I’ve let my child find [information] on their own, and whenever they want to share things with me, we talk about it…the conversations are started by the child, because that seems to work best…[My children] don’t really want me to teach them. They want to come into conversations with me as more of an equal having their own knowledge…they are, to some extent, part of a different culture than we are, and they like to educate us about that culture. That always goes better than me trying to hand them resources. (ID15)

In contrast, many youth participants described not wanting to be the sole source of information for their parents on gender and sexuality-related topics. There was a tension between parents believing that it was most effective to let their child educate them about these topics, and youth wishing their parents had sought out other sources of information. One non-binary youth believed that parents should “look [information] up on their own after their kid comes out” and described feeling “upset” that all of their parents’ knowledge about transgender health and identity had to come from them (ID10). Multiple youth participants discussed the emotional labor that was inherent to educating their parents on these topics:

It's important for parents and other adults in children's lives to understand [transgender health], and how [parents] can be supportive, so that youth don’t have to bear the burden of doing all the emotional labor of educating everyone in their lives when they’re still figuring this out. (ID3)

A healthcare affiliate emphasized that youth should not be responsible for educating their parents, because when parents are already informed, they are better able to fulfill their responsibilities as parents in sexual health discussions, such as talking with their child about setting boundaries in relationships and safer sex practices:

My goal with parents is to get them to a place of decreased intimidation about [transgender topics] and not relying on their kids to be the expert about being transgender. The kids are just being kids. They happen to be trans or gender nonconforming. Doesn’t mean they know the lay of the land. So I want parents to recognize that they still have a parental responsibility. And if their kids don’t know, it’s still their responsibility to step in as much as they can. (ID20)

Overall, youth, parents, and healthcare affiliates agreed that parent–child conversations around puberty, sexual health, and gender serve a dual purpose as an important means of (1) social support for TNB youth and (2) promotion of safety across multiple domains, including physical safety against STIs, unwanted pregnancy, and sexual violence, as well as emotional safety through healthy romantic relationships. One healthcare affiliate emphasized the importance of parental confidence in topics about sexual health and gender identity in order to encourage safe and affirming romantic and/or sexual relationships for their child.

For parents, the more you step into this and the more confidence you have [in leading these conversations], the better you’re going to be able to serve your child. Hopefully, fit that into the same [conversations] that parents of [cisgender] kids also need to [have]: talk about safety and not just safety with respect to sex but safety with navigating relationships, safety from decreasing situations where sexual assault could happen, that kind of thing. (ID20)

Youth participants echoed many of these sentiments and believed that their parents would have been better able to support them had they sought out or had access to more transgender-specific sexual education resources. One youth shared that their parents “were the people I trusted most, and I wanted to talk to them, but they didn’t know anything either” (ID9). Parents, youth, and healthcare affiliates all believed that baseline parental knowledge about TNB sexual health and confidence played a role in the effectiveness of these conversations.

### Barriers to Accessing Information

Youth, parents, and healthcare affiliates all discussed varying levels of concern about the reliability and accuracy of information they found online. Healthcare affiliates noted that youth tended to be more trusting of information they found online, whereas parents “tend to be pretty skeptical of all online sources” (ID17). This resulted in parents relying on their child as their primary source of information, which was coming primarily from online sources that the parent did not trust.

Parents who described seeking independent resources often struggled to find credible sources of information. This was particularly difficult for parents once their child began seeking medical interventions, such as hormone replacement therapy (HRT).

When he started saying “I want to go on T [i.e., testosterone]; I want to have top surgery” then we were like, “Where’s the research?” And it was incredibly hard to find…I was literally at the library, trying to look up medical articles, and I can hardly read them. But I just wanted to know, what are the side effects, just kind of the basic stuff before I’d even gotten to talk to a doctor. (ID15)

One parent expressed concerns about relying exclusively on physicians, particularly in the context of information on fertility preservation. She recalled desiring information about HRT and the fertility “decisions you need to make together” (i.e., egg/sperm freezing), because “some doctors are better at talking you through all that than others…and you may end up talking to a doctor who kind of blows by that” (ID15). This parent also noted that because youth are “pretty adept at researching online,” they often have more information than the parent, which can create a knowledge imbalance. For example, in conversations about medical interventions the child is hoping to access, parents were concerned that their child may not share information about potential complications or other aspects they know their parent would be apprehensive about.

### Recommended Curricular Content for Parents of TNB Youth

Almost all participants agreed that TNB youth and their parents have fundamentally different educational needs around the topics of gender and sexual health and believed it was important for separate resources to be specifically designed for parents of TNB youth. Participants recommended the following six content areas be included in a gender and sexual health curriculum aimed at parents of TNB youth: basic information on gender identity and trans-competence, diverse narratives of TNB experiences and identities, gender dysphoria, non-medical gender-affirming interventions, medical gender-affirming interventions, and resources for peer support.

Participants discussed multiple reasons for educating parents separately from youth. Namely, for parents, conversations related to sexual health were often eclipsed by conversations about gender identity and safety of gender-affirming interventions. Parents tended to be more concerned with long-term considerations of transitioning (e.g., fertility preservation; health risks associated with HRT; concerns about future desistence of TNB identity, or that identifying as TNB is a “phase”), whereas youth were more concerned that their parents understand the breadth of diversity within gender and sexuality and have a broad understanding of transition-specific concepts (e.g., HRT, binding) in order to support them throughout their transition.

In addition, participants believed that separate youth and parent curricula would help parents process their fears and other emotions separately from their kids, as well as allow them to learn how to best support their child:

I had to work on a lot of shame in my own self. Even though I wasn’t—I really honestly had no problem with my kid being trans. I had to work on the grief of letting go of the future I had pictured my kid having. And it changed our relationship. I used to be in the little club of people known as moms who have daughters. I’m not in that club anymore, exactly. So, that kind of work, I would not want parents to be doing in front of their kids. That’s why I’m like, “They [i.e., parents and their TNB children] have separate needs.” (ID12)

#### Information on Gender Identity and Trans-Competence

Almost all youth and parents described the need for a curriculum to address basic concepts about gender identity and sexual orientation. Parents often described the revelation of learning that sex assigned at birth is a separate concept from one’s from gender identity, and different still from sexual orientation. While a number of parents believed they had a strong understanding of these concepts at the time of being interviewed for this study, they often expressed that having this information earlier would have been helpful to “give them all of the pieces so they can start to see how they fit together…sexuality, gender identity, [and] gender expression” (ID16).

Other parents expressed continued confusion about the differences between gender and sexuality, and discomfort with concepts related to TNB identities:

[Resources I’ve seen] don’t really talk much about the different genders that you could pick to be, or not *pick* to be, but that you *could* be. Like I know that you can be transgender, or you can be gay, or bi, or…Well, I guess, no, that’s different because your sexuality and your gender are different. And they don’t tell you that either. (ID13)

Many participants also felt it was important to consider how non-binary and intersex identities and experiences fit in their framework for understanding gender and “biological” sex. A number of participants specifically identified knowledge deficits about non-binary gender identities and advocated for curricular inclusion of this population: “it shouldn’t make assumptions about two genders” (ID12). In addition, several participants explained how learning about intersex identities helped them to understand how even sex is not binary: “Not everyone is born completely female and completely male. I don’t think people understand that there are some people who are XXY…These are not even things that have ever come across their view of the world” (ID14).

One parent revealed the damaging impact of her anti-transgender bias on her ability to have supportive conversations with her child. Specifically, this parent described disagreeing with her transmasculine son about the validity of non-binary identities, which led to tense, unproductive conversations between them.

Whenever I talk to my son, it sometimes can be difficult because I feel like he’s very judgmental of me…If I say, “it’s going too far to expect people to call somebody that is gender fluid ‘they’ or nothing at all, then how are you supposed to address that person?” And he thinks that is just wrong, and I’m just not right because I say that. And that makes me feel very judged. It makes me feel like he thinks I’m a bad person. It makes it very difficult [to have conversations]. (ID13)

Consistent with our previous findings, most parents recognized the importance of using gender-affirming and trans-competent language when discussing anatomy and sexual health with TNB youth. One parent noted, “It seems [like] unnecessary, complicated language…[but] I think understanding that this is not just what people dismiss as politically correct language, this is part of someone’s identity that you have to respect” (ID15).

Beyond understanding basic concepts and terminology related to gender identity and sexual orientation, some parents described being concerned that their child’s gender identity is a choice or could be a phase (“My husband wanted to know things like…‘what if my kid changes their mind?’”), while other parents expressed frustration that other adults disregard the validity of their child’s TNB identity.

I think the biggest [myth] is that people think this is a choice that a kid makes, to be trans…They’re thinking that you’ve got a teenager [that] just decided one day that they wanted to be trans. I think a lot of parents who don’t accept that their kid is trans, it’s because they feel that their child has decided they want to be this, and it’s just a fad that’s going to pass. I mean, I wondered that too when we first faced this, but I’ve since learned that no, that’s not the way it goes. (ID16)

This sentiment was strongly echoed by the youth participants, who felt that parents should understand that their gender identity was not a choice. One youth stated that many of their family members believe that they could choose not to be TNB, which was very upsetting for them: “[My] family members…think ‘you don’t have to choose to be this’” (ID10). This was also mentioned by healthcare affiliates, who regularly field questions about how often TNB youth “change their minds.”

From the parent’s perspective [we get asked], “Will they change their mind once we start hormones?” [There] is this misconception that this young person–how do they know what they want to be at this age? And again, “Will they change their mind? Because we’re human we always change our mind.” So I have to say that we don’t have a whole lot of evidence that people do change their mind; identity usually doesn’t change. (ID21)

#### Inclusion of Diverse Narratives

Youth and parents desired exposure to a diverse set of narratives and representations of TNB individuals and their experiences. Parents believed this could be reassuring to other parents, and potentially lessen concerns about obstacles and hardships their child may face. Youth believed the use of narratives and positive representations of TNB people would normalize the experiences of TNB people and expose parents to the broad range of identities and life paths that are possible for TNB people. For example, one parent described how personal story-telling has helped her build empathy, understanding, and hope for her child:

[When I hear] stories about people who don’t identify as one of the binary sexes, male or female, and these people sharing personal stories…[it] give[s] me insight as to how my child might be feeling. And also, it helps me understand better how to respond to people who don’t identify in the traditional way we’ve been taught to see gender. And I think that’s incredibly helpful too because…we’re not our sexuality. We’re people, and our sexuality is one part of our identity. And people sharing their stories about how it is to be seen as different and how they perceive barriers, the things that make them sad, the things that cause them pain, and then how they triumph through those challenges and find people who support them. I really enjoy those stories because it gives me hope that my own child’s going to be okay. (ID15)

Another parent emphasized the importance of holistic representation and inclusion of TNB people across life stages:

First of all, just feature trans kids, and trans parents, and trans everybody…It should have all that kind of–that lens of equity to look at the entire thing holistically. But it also should just normalize the experience. It should also leave a lot of spaciousness for the incredible diversity of the experience. (ID12)

Some youth emphasized the importance of narratives that exemplify the difference between gender and sexual orientation (e.g., that a transmasculine person may identify as gay). One parent noted that in order to be a supportive parent, it was important for her to understand these differences and be comfortable with her child discovering new aspects of his identity.

I have a trans son, and I have no idea who he’s really attracted to. And I’ve asked him, but I’m not sure he really understands at this point. Parents need to understand maybe that it is a fluid sort of thing for these kids, and maybe you just need to give it time until things sort out…We’re always taught the binary system that there are males and females, [and] parents are going to have a difficult time, I think, understanding the intricacies of the world in which that is not true. (ID16)

#### Gender Dysphoria

Most youth participants talked in detail about experiencing gender dysphoria and the impact it had on their well-being, both in general and specifically for their sexual health. The term “gender dysphoria” defines both a medical diagnosis, as well as an embodied experience, and can be described as an “incongruence” between one’s gender and sex assigned at birth and may be “associated with clinically significant distress or impairment in social, school, or other important areas of functioning” (American Psychiatric Association, 2013). Importantly, not all TNB people experience distress related to incongruence between their assigned and identified gender, but some do, especially in the absence of gender-affirming interventions that align their body more closely with their gender*.* For youth in our study, gender dysphoria was particularly relevant in the context of puberty, a time that many youth and healthcare affiliates described as a period of worsening gender dysphoria and heightened distress.

Youth participants felt that gender dysphoria was an important topic for their parents to learn about. One youth shared that they desired a curriculum that teaches parents in “how to affirm [their child] or how to help them with [gender] dysphoria” (ID11). Healthcare affiliates in particular believed that it was important for parents to understand that puberty can increase gender dysphoria for their children.

For a lot of people, the case is that puberty worsens gender dysphoria, right? So helping parents understand that and how that might fit into their child’s story when [parents say], “Oh, I don’t know how suddenly, two years ago, their grades dropped, and they got really upset, and they lost all their friends, and also puberty.” And I’m like, “Okay, how do we connect those dots?” Because if you’re not looking for it, you don’t. (ID17)

Only one parent participant explicitly referenced gender dysphoria, though many described interactions with their child that were likely related to gender dysphoria.

There’s also the gender dysphoria piece, so [that] I understand why [my son] wants to wear [a binder]…something that makes really clear, this why your child is going to want to do it. I don’t think you can have a curriculum that doesn’t include [dysphoria], so that we [i.e., parents] realize it’s real and take it seriously. (ID15)

#### Non-Medical Gender-Affirming Interventions

Youth and parents advocated for the inclusion of basic information about non-medical gender-affirming techniques, such as binding, packing, stand-to-pee devices, tucking, electrolysis, voice therapy, bras, make-up, and more (Table 3). In general, youth were fairly informed about what options were available to them (primarily through online research) and felt that their parents knew little about these topics. Some youth believed parental access to this information would be particularly important for ensuring the safety of their children when using these aids:

When I was binding unsafely, my mom didn’t really know about binding safety. If I said, “No, it’s fine,” she just had to take my word for it. Which was convenient for me, if I wanted to keep binding, but for safety reasons, I think it’s important that parents can access the information on their own, just because not everyone else is going to be upfront about it. And it’s easier for my mom to point out to me that I need to stop binding because it's not safe than for me to make that decision on my own. (ID10)

**Table 3:** Terms and descriptions for non-medical gender-affirming techniques

Term	Description
Binding	Using constrictive materials to flatten breast tissue and create a flat-appearing chest
Packing	Wearing padding or a penile prosthetic in the pants to create a bulge
Stand-to-pee device	Device that aids a person in urinating while standing upright; some may double as packers (see above)
Tucking	Pushing the testes into the inguinal canal and securing the penis back between the legs to minimize the contour of the genitals
Electrolysis	Hair removal therapy that can permanently remove undesirable facial and/or body hair
Voice therapy	Therapy provided by speech language pathologists to assist individuals in adjusting their pitch and intonation to more closely match their gender expression

One parent described learning about safety concerns around binding from her child’s physician and explained that understanding binding helped her comprehend her child’s desire for top surgery.

[I] didn’t realize until we went to speak with the doctors at the gender clinic that some kids wore their binders 24 hours a day. Even just the amount of anxiety my son can have over not having a clean binder available on Monday morning, that’s enough that you might not be able to go to school. And that sounds crazy to a [cisgender] parent, but it’s not…I think one reason that top surgery really makes sense to me is because the alternative is a binder. And the fact is, if you can move and breathe, you’re just a lot better off than if you can’t. I felt differently about [top surgery] after learning more about the binder. (ID15)

Supporting youth as they explore their gender presentation, particularly through clothing, was a common theme. One parent noted that parents should understand why attire matters so much to their TNB youth:

For a parent, [gender expression] can translate as unreasonable concern over clothing, which we think of, I think, as something teenagers are too obsessed about. But this is different…[for trans youth] this is not a status thing [or a] competing with my classmate thing. This is who I am. (ID16)

#### Medical Gender-Affirming Interventions

Puberty blockers (gonadotropin-releasing hormone agonists), HRT, and gender-affirming surgeries were discussed by all participants and identified as essential content to include in a curriculum for parents of TNB youth. Although not every TNB individual will desire all (or any) of these medical interventions, almost every participant believed they were essential topics about which parents should be familiar. Parents were more concerned about possible long-term complications (health risks associated with HRT, fertility preservation, surgical outcomes), whereas youth were more concerned that their parents be informed enough to support them as they navigated the medical system.

One parent described concerns that arose during the process of their child starting testosterone therapy and developing clitoromegaly—a common and often desired effect of initiating testosterone:

Going through the consent form on [testosterone] and going through what the possible side effects are, we do kind of slide past what happens to your genitals when you’re on T. I mean, it was kind of mentioned, but I’m thinking: What are the ramifications of that? Are we going to need different kinds of underwear? Do you get very sensitive? Do you have pain? (ID15)

Parents believed it was important for other parents to have access to information on medical interventions so they can be on more equal footing with their children, who are often more aware of different intervention options, and thus be better able to make decisions together.

I think it would be really helpful just to have the basics of [HRT]…this is information the kid is likely to have well before the parent, and the parent will be catching up on. Having some basic information in there about how [exogenous hormones] work, and what potential ups and downs are, pros and cons, and things that you need to think about–decisions you need to make together. (ID15)

This informational imbalance between parents and youth was also observed by healthcare affiliates who noted that parents were often less informed than their child.

Often our teens’ questions are more detailed because they’ve done more research, so they’re like, “Well, what’s the difference between the estradiol patch form, or injection, or oral, or sublingual?” and parents are like, “What?” (ID17)

Upon first learning that their child was interested in HRT, many parents recalled having concerns and misinformation about the dangers of hormone therapy. Accurate information about HRT would help parents dispel myths about health risks.

I think the biggest [myth I heard] was about hormones being really dangerous. And it was interesting because I had been talking to friends my age who had been saying that they had heard anecdotally that there was a lot of organ failure in people our age who’ve been on hormones for 30 years, and that it was really hard on your liver and all this sort of thing. And when I started looking into the research, I did not find that, and then, when we talked to the doctors, they also said that that was not true....but I really believed that the side effects were a lot worse than any of the research has shown. (ID15)

Parents and healthcare affiliates both emphasized that fertility concerns are often of primary importance to parents when considering HRT, while youth were perceived by both providers and parents as being less concerned with fertility.

Parents tend to be concerned with the long-term consequences like fertility, which often teens are like, “I’ve already made decisions about that,” for better or for worse. And although we do have a lot of teens that acknowledge like, “Yes, that could change, and here’s what I know now,” teens are often more concerned with timeline. You are living in a body that is very uncomfortable, so of course, you’re like, “When can this [i.e., HRT] happen?” (ID17)

Some parents took the view that their adolescent children lacked the capacity to make an informed decision about fertility preservation and believed that having accurate information would facilitate important conversations with their child:

A kid at 14 cannot decide if they want to have children someday…So you have to be able to try to make a decision about how you’re going to plan for the future together, and those are hard conversations to have, but I think the more information you have, the better off you are. (ID15)

Few youth participants discussed fertility preservation or long-term outcomes of HRT during their interviews. Instead, most youth participants emphasized that if parents were more informed about the effects of HRT, they would better be able to support their child.

[Parents should have] information on how to be an ally and how to support your child if they’re going through [HRT]…A lot of parents just are completely at a loss on how to support their child…because they don’t have the information on how to do that and what’s going to happen. …I feel like it would be [great if a] resource [could explain] those changes, look at diagrams, talk about the changes, [explain] on how to be an ally, and how to support your child, and [say] “Here are the changes you’re going to see. Here are the things that are probably going to make you uncomfortable and how to come to peace with that.” How to be supportive. (ID9)

Regarding surgery, parents found that when they had more information about surgical options, they were able to be understanding about their child’s decisions and more supportive overall. One parent described the topic of surgery coming up for the first time at a gender clinic appointment; their child had been afraid to mention it sooner for fear of “piling too much on [me] at once.” This parent believed that having “some basic information about [surgery] upfront would make sense” and also provide context for discussion of other non-medical interventions such as binding. The parent stated that they felt better about top surgery after “learning about it more and doing exactly what my kids do, which is going on Instagram and finding people who’d had top surgery and looking at the pictures they posted of themselves living their life without boobs and being very happy about it…At first I was really kind of worried. Now I’m thinking more about the aesthetics” (ID15).

#### Resources for Parental Peer Support

For some parents, peer support was cited as something that either has been or would have been tremendously positive for them. Parents found that peer support was helpful for seeking out advice, processing complex emotions, and feeling less alone in the experience of parenting a TNB child. Many parents felt that this would be particularly helpful in the context of dealing with negative emotions such as guilt, grief, and shame—emotions that they did not want to process around their child.

…[T]here’s a place for [parents] to be validated that it’s a common experience to have grief. It’s a common experience to feel disoriented…and saying everyone’s experience is going to be different, but here are some things that other parents of trans kids have experienced that you might find reassuring to know that you’re not alone in this. (ID12)

Another parent (ID16) described a beneficial experience meeting other parents of TNB youth at a conference: “It was heart-wrenching to listen to these [parents’] stories. But it was very good. It was really therapeutic for me to really understand that I wasn’t alone.”

Several healthcare affiliates noted the importance of parents having access to peer support. In addition to accessing support to process their negative emotions, this type of community can help strengthen parenting skills and facilitate increased parental confidence in leading conversations in topics uniquely affecting transgender youth, such as issues of identity disclosure:

[It’s important to] help these caregivers of these kids feel more confident to step into conversations. Things like disclosure, and safety, and privacy…Families [must] work together and consider, “How do I navigate this? Do I not engage physically with somebody [in a romantic way]? People don’t know [about my transgender identity], but maybe I need them to know.” All of these are worthy conversations [for parents] to have [in] a society that is not yet confident or familiar enough with the experience of trans kids. (ID20)

## Discussion

Conversations between TNB youth and their parents about puberty, sexual health, and gender can be an important means of social support for TNB youth and also promote safety and healthy relationships for TNB youth. These conversations and their role in providing education and support to TNB youth may be increasingly important in light of expanding legislative attacks on the rights of transgender youth (Kidd et al., 2021). Parents, youth, and healthcare affiliates in our study all believed that baseline parental knowledge about TNB sexual health, and parental confidence in leading these conversations, plays a role in their effectiveness.

Notably, all participants agreed that parents have different informational needs than their TNB children and advocated for a curriculum tailored to parents of TNB youth. In parent interviews, conversations about sexual health were described as frequently being eclipsed by discussions of gender identity and safety of gender-affirming interventions. This was sometimes a source of tension, as parental focus was often on long-term considerations (e.g., fertility preservation, health risks of HRT), whereas youth were more focused on their parents understanding basic concepts about gender identity, sexuality, and transition-specific concepts (e.g., HRT, binding) in order to support them and help them navigate medical systems.

Our findings emphasize that greater efforts are required to meet the educational needs of parents of transgender youth. A lack of parent–child conversations, or conversations that youth believed were unsupportive, often led to feelings of resentment. These findings are reflected in the literature on the importance of parent-adolescent sexual communication, although almost all of the existing studies focus on non-LGBTQ youth. For example, one recent meta-analysis found that increased communication between parents and adolescents about sexual health is associated with increased use of contraceptives and condoms (Widman et al., 2016). Another study examining parent–child communication about sex for young men who have sex with men found an association between comfort with parent–child sex communication and current PrEP use (Flores et al., 2020). This is particularly relevant given sexual health disparities faced by transgender youth, such as increased risk of acquisition of HIV and other STIs (Reisner et al., 2019). Though some parents attempted to address STI prevention in their parent–child conversations, they did so through an assumed cisgender/heterosexual lens, which may not address TNB youth’s specific experiences.

In our study, parent–child conversations about puberty and sexual health that took place prior to youth coming out reflected similar themes to those found in the literature related to non-LGBTQ youth (Flores & Barroso, 2017; Jerman & Constantine, 2010; Pariera, 2016). There was a wide range of styles of parental communication, perceived effectiveness, and youth and parent comfort levels.

However, while parent-led conversations about puberty and anatomy were common for most of our study participants, the reported role-reversal in which youth teach their cisgender parents after coming out may be unique to sexual and gender minority youth. This is particularly important in light of the findings that LGBTQ youth have unique sexual educational needs and are often dissatisfied with the puberty and sexual health information available to them; therefore, parental conversations have the potential to fill in some of those gaps in school-based sources of sex education (Dewaele et al., 2017; Haley et al., 2019; Poteat et al., 2019; Savin-Williams & Diamond, 2000; Sexuality Information & Education Council of the United States, 2015).

Instead, many youth participants felt frustrated that they were constantly in the role of teacher when it came to discussions of gender with their cisgender parents, including during sexual education conversations. Interestingly, there was a disconnect between parental perceptions and youth experiences, in which parent participants often believed that youth preferred being in the role of teacher. Healthcare affiliates also echoed the importance of parents being able to productively contribute to conversations with their TNB child, emphasizing that this would help parents feel more comfortable talking about general topics (e.g., healthy relationships and boundaries), as well as topics more specific to the TNB experience (e.g., when and how to disclose TNB identity to peers and partners). Parental support has also been shown to correlate with increased disclosure of transgender identity to healthcare providers, something that is essential to ensuring TNB youth are able to access affirming healthcare (Sequeira et al., 2020).

In general, participants believed that a sexual health curriculum for TNB youth and their families should contain separate content for parents and youth because of different educational needs. This is an important finding, as there is limited research examining whether other groups of youth and their parents believe parents have distinct educational needs compared to their children. It is interesting that despite the study focus on sexual health, most themes raised by parents and youth alike related to puberty, dysphoria, and medical/non-medical gender-affirming interventions. Based on this, we posit that in order for sexual health to be effectively discussed between parent and child, parents’ first need is to achieve a baseline level of knowledge and comfort related to discussing the above topics. In two-parent households, providing parents with a source of accurate information can also help ensure consistent messaging and support between both parents, as differing levels of parental support can be an additional stressor for TNB youth (Pullen-Sansfacon et al., 2020; Wilson et al., 2016).

For most of our participants, conversations about sexual health were often overshadowed by parental questions about gender identity and medical interventions. Thus, when identifying ideal content for parents of TNB youth, most respondents prioritized teaching the basic concepts of gender identity, sexual orientation, non-binary identities, and gender-neutral language. Educating cisgender parents about these topics may help to combat “gender-stereotypical attitudes” toward parenting, which has been shown to be associated with increased behavioral and emotional challenges for gender non-conforming children (MacMullin et al., 2021). Dispelling the idea that transgender identity is a choice or phase was another recurrent theme for participants, one that parallels the finding that parents of LGBQ youth often also often believe that LGBQ identity is a phase (Burton & Lothwell, 2012). While it is true that sexual orientation and gender identity may change and evolve over time, many participants took issue with the idea that their TNB identity was transient and that they would eventually identify as cisgender.

A number of youth and parents believed that inclusion of diverse narratives about TNB individuals would be a useful curriculum feature. Youth were particularly interested in narratives that showed the range of TNB experiences, including examples of diverse and multiple marginalized identities and a range of life experiences. They believed this would help normalize TNB experiences and expose parents to the breadth of life paths possible for TNB individuals. Parents also believed narratives would help soothe some of their worries about their child’s future. These narratives can be useful for balancing an appropriate understanding of the specific challenges often faced by TNB youth (e.g., risk of bullying, mental health challenges) without catastrophizing their future. Studies have shown that parents of transgender youth require positive portrayals of transgender individuals and communities in order to soothe concerns about their child’s future as well as to inform parents that gender variance is part of the normal variety of life (Riley et al., 2013a, 2013b). Including these stories would be an innovative way to expose parents to positive representations of TNB people and simultaneously challenge binary assumptions about gender and sexuality. These representations could come from individual youth stories or other media sources, many of which emphasize the importance of story-telling and positive representation of TNB youth (Meadows, 2018; Travers, 2018). Ultimately, access to this type of information could help lessen the burden on youth to teach their parents, something that can be particularly stressful if parents are still coming to terms with their child’s identity.

Healthcare affiliates and youth, in particular, advocated for a curriculum to include information about gender dysphoria, its relationship to the onset of puberty, and how to support a person experiencing dysphoria. Numerous studies have shown that in TNB youth with dysphoria, social support and/or access to medical interventions for TNB individuals who desire them is highly correlated with better mental well-being (e.g., decreased depression/anxiety, improved body image/satisfaction, self-reported quality of life) and decreased lifetime incidence of suicidal ideation (Cohen-Kettenis et al., 2011; de Vries et al., 2011, 2014; Nakatsuka, 2012; Tordoff et al., 2022; Turban et al., 2020). Thus, our study participants believed that this information would provide important context for the distress often experienced by youth during puberty and help parents understand why youth may desire access to gender-affirming interventions.

To complement this information, both parents and youth felt it was important for parents to learn about non-medical gender-affirming techniques (e.g., binding, tucking) and their relevant safety concerns. For example, a large study on binding experiences among adults who were assigned female at birth found that while participants overwhelmingly felt that binding improved their mental health, nearly all (97%) reported at least one negative physical health outcomes attributed to binding (Cohen-Kettenis et al., 2011). The most common reported outcomes were back pain, overheating, chest pain, shortness of breath, itching, bad posture, and shoulder pain; negative outcomes were associated with increased frequency of binding (Peitzmeier et al., 2017). When parents are informed about these interventions, they can more easily understand why youth with gender dysphoria need access to them and may be better equipped to provide guidance in cases of unsafe implementation (e.g., sleeping in a binder).

Participants also thought it was important to address common parental concerns about medical gender-affirming interventions, including the long-term effects of exogenous hormone use and the impact of medical interventions on fertility. Our findings related to differential parent and youth concerns about fertility are consistent with existing literature on TNB adolescents. For instance, one study assessing transgender youth and parent attitudes toward fertility preservation and HRT found that many youth wished to parent a child someday, but few explicitly desired a biological child (4%) (Strang et al., 2018). A significant portion of youth felt pressure from their family to have a biological child (25%) and many youth wondered (50%) or did not know (20%) if their feelings about biological children would change in the future. Parents were more concerned about their child changing their mind and later wanting biological children, and 21% stated that they would be disappointed if their child could not have biological children. Nearly all parent participants in this study emphasized the importance of learning how HRT may affect fertility. Another study assessing fertility decision-making among transgender young adults identified shortcomings in healthcare providers’ fertility counseling—such as information that was incomplete and affected patients’ decision-making satisfaction (Chen et al., 2019). This further supports our finding that fertility education is important to parents of TNB youth and may assist parents in facilitating more informed conversations about healthcare decisions and future fertility options with their child. It is also important to note that few insurance plans currently offer significant coverage for fertility-related services; therefore, there are many barriers around access to these services, particularly for youth and families with lower socioeconomic status (Kyweluk et al., 2019).

Finally, many parents and healthcare affiliates emphasized the importance of peer support for parents. Some participants believed that a dedicated educational curriculum for parents would facilitate protected space for them to process emotions such as grief and shame that may be harmful for their children to witness. This could be helpful to mitigate the long-term harms that have been well-demonstrated as a result of adverse parental responses when youth first come out (Newcomb et al., 2019, 2020; Saltzburg, 2004). Family rejection or perceived family rejection has been shown to correlate with higher rates of negative health outcomes as well as internalizing disorders (Klein & Golub, 2016; Olson et al., 2016; Valente et al., 2020).

### Limitations

There were a number of limitations to this study. The sample size was small, and all youth and parent participants were white and resided in the Seattle-area, in a state where little to no anti-trans legislation currently exists. Thus, data are likely not generalizable to other geographical locations or racially diverse populations. Additionally, most parent participants were generally supportive of their child and the youth participants had access to gender-affirming care. Therefore, our findings are likely not applicable for less supportive parent populations or youth who do not have access to gender-affirming care. All parents interviewed were cisgender women, and it is possible/likely that interviews with parents of other gender identities would have generated different results. Future work should deliberately explore these perspectives. In addition, all parent participants were parents of TNB youth assigned female at birth, and we may have obtained significantly different responses from parents of TNB youth assigned male at birth. Transfeminine youth were underrepresented among youth participants, which likely influenced the topics of interest in participant interviews and limited generalizability of findings. However, a strength of this study is that it was not limited to parent perspectives; youth and healthcare affiliates provided insightful perspectives on parents’ knowledge gaps, and these two groups are perhaps better equipped than parents themselves to identify such knowledge gaps. Put another way, it requires a degree of self-awareness to “know what you don’t know,” which parents may not always have. This makes the perspectives of youth and healthcare affiliates so valuable.

Additionally, due to the nature of agreeing to take part in this study, the sample of parents were likely more supportive of their TNB children, may have been more knowledgeable about sexual health and other relevant topics and likely had more access to gender-affirming healthcare compared with the broader TNB parent community; their opinions may therefore differ from parents who are less supportive, less knowledgeable, or who experienced less healthcare access. In spite of these limitations, we believe this paper represents a starting point in the exploration of a critical topic. Additional research is recommended to explore the needs of diverse parents of diverse TNB youth, to confirm and/or expand upon themes detected in this study.

### Conclusion

This study represents a first step toward exploring parent knowledge gaps regarding gender and sexual health for TNB youth, and identifying specific educational content deemed important by youth, parents, and healthcare affiliates to fill these gaps. Parents of TNB youth, TNB youth themselves, and medical providers all felt it was critical for parents of TNB youth to have access to information to help them support their child’s sexual health as they navigate the social, psychological, and medical aspects of transitioning to their affirmed gender identity. Findings specifically favor the development of a tailored educational curricula to equip parents in conducting informed, non-judgmental conversations about sexual health, as well as in more broadly supporting their children as they navigate the experience of being transgender or non-binary. These findings are critical given the increasing barriers that exist for TNB youth to access affirming sexual health information and care. Future research should expand this work to explore perspectives of a broader group of TNB youth and parents with a variety of genders and geographical, racial/ethnic, and socioeconomic backgrounds.

## Supplementary Information

Below is the link to the electronic supplementary material.

Supplementary file1 (DOC 16 KB)
