# Supplementary material for: Gender and Sexual Health-Related Knowledge Gaps and Educational Needs of Parents of Transgender and Non-Binary Youth
Source: Arch Sex Behav. 2023 May 26;52(5):2185–203. doi: 10.1007/s10508-023-02611-9 (PMC10322789; doi:10.1007/s10508-023-02611-9)
Supplement: Supplementary file 1 — Supplementary file1 (DOC 16 KB) [file 10508_2023_2611_MOESM1_ESM.doc]

**Supplement 1** - Interview script topics and prompts

| **Topic** | **Example Prompts** |
| --- | --- |
| **Sources of Information**  (all participants, except where noted) | - (Youth/parents only) Where did (you/your child) get information about puberty, sex, and sexual health when (you/they) were growing up? - (Healthcare affiliates only) What types of resources have you patients accessed related to puberty and/or sexual health? (eg. conversations, doctors, in school, from peers) - (Youth/Parents only) Tell me about sex education at (your/your child’s) school. What grade was it in and what content was covered? - What topics does/did it cover? - What information does/did it not cover? - Does/did the information source talk about transgender or gender-diverse identities? - Do you feel like it met (your/your child’s/your patient’s) needs? If not, what was missing? - How did receiving this sex education program make (you/your child/your patient) feel? - (Youth only) Did your doctors//peers/parents ever talk to you or provide information to you about sexual health? |
| **Parent-Child Conversations** (youth and parents) | - At what age do you feel like it is important to talk to trans and GNC youth about puberty? Sexual health? Healthy relationships, and/or boundaries? - Do you feel like your conversations met your child’s needs? If not, what was missing? |
| **Content**  (all participants) | - What type of information do you think is important to share with trans and GNC youth about puberty and sexual healthy? Why? What type of information would be important to share with parents? eg.   - STIs, HIV/AIDS, testing   - Condoms, PrEP and PEP   - Pregnancy, contraception   - Gender-affirming choices such as binders, packers, bras, and makeup   - Gender-affirming choices such as HRT, surgeries, puberty blockers   - Puberty education   - Sex acts, attraction, arousal   - Sex toys   - Identity (gender identity sexual orientation, etc.)   - Dating, health relationships, boundaries, consent, disclosure   - Access to clinical care, sexual healthcare, finding and talking to doctors - What information is least important? - What type of content is most challenging for you to find? What type of information do you still want to know more about? - What type of misinformation or myths have you seen or heard related to sexual health in general? What about related to transgender sexual health specifically? - What mode do you believe would be ideal for delivering content to youth and parents (eg. online, in person)? |
| **Importance**  (all participants) | - What type of important take-aways or information would you want (you/your child) to have after a conversation/exposure to puberty or sexual health information? |
| **Interactions with Patients and their Parents**  (healthcare affiliates) | - In your [practice/clinic], can you describe what your role is in working with patients? How often do you see patients who are TNB youth or their parents? - What are common concerns or questions you get asked by patients? Parents? How does this differ? - What are the largest knowledge gaps, or examples of misinformation that you hear from your patients and their parents? - What type of information do you think is most important to share with trans and GNC youth about puberty and sexual health? Why? - What type of information do you think is most important to share with parents? Why? |
